# Excellent catalysis of Mn_3_O_4_ nanoparticles on the hydrogen storage properties of MgH_2_: an experimental and theoretical study†

**DOI:** 10.1039/d0na00137f

**Published:** 2020-03-09

**Authors:** Liuting Zhang, Ze Sun, Zhendong Yao, Lei Yang, Nianhua Yan, Xiong Lu, Beibei Xiao, Xinqiao Zhu, Lixin Chen

**Affiliations:** School of Energy and Power, Jiangsu University of Science and Technology Zhenjiang 212003 China; Institute of Nuclear Physics and Chemistry, China Academy of Engineering Physics Mianyang 621999 China zhuxinqiao@zju.edu.cn +86 17738406685; State Key Laboratory of Silicon Materials, Department of Materials Science and Engineering, Zhejiang University Hangzhou 310027 China lxchen@zju.edu.cn

## Abstract

Recently, transition metal oxides have been evidenced to be superior catalysts for improving the hydrogen desorption/absorption performance of MgH_2_. In this paper, Mn_3_O_4_ nanoparticles with a uniform size of around 10 nm were synthesized by a facile chemical method and then introduced to modify the hydrogen storage properties of MgH_2_. With the addition of 10 wt% Mn_3_O_4_ nanoparticles, the MgH_2_–Mn_3_O_4_ composite started to release hydrogen at 200 °C and approximately 6.8 wt% H_2_ could be released within 8 min at 300 °C. For absorption, the completely dehydrogenated sample took up 5.0 wt% H_2_ within 10 min under 3 MPa hydrogen even at 100 °C. Compared with pristine MgH_2_, the activation energy value of absorption for the MgH_2_ + 10 wt% Mn_3_O_4_ composite decreased from 72.5 ± 2.7 to 34.4 ± 0.9 kJ mol^−1^. The catalytic mechanism of Mn_3_O_4_ was also explored and discussed with solid evidence from X-ray diffraction (XRD), Transmission Electron Microscope (TEM) and Energy Dispersive X-ray Spectroscopy (EDS) studies. Density functional theory calculations revealed that the Mg–H bonds were elongated and weakened with the doping of Mn_3_O_4_. In addition, a cycling test showed that the hydrogen storage capacity and reaction kinetics of MgH_2_–Mn_3_O_4_ could be favourably preserved in 20 cycles, indicative of promising applications as a solid-state hydrogen storage material in a future hydrogen society.

## Abstract 2

Metallic Mn reduced from Mn_3_O_4_ coated on the surface of MgH_2_/Mg weakened the Mg–H bond and helped to preserve the cycling properties.

## Graphical abstract

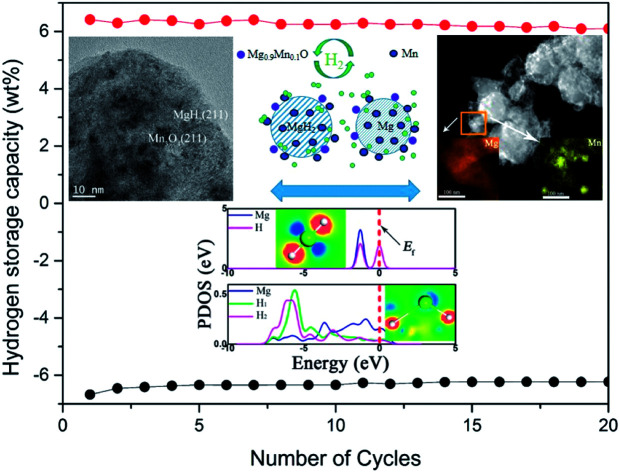

## 1. Introduction

Faced with a global energy crisis and environmental issues, the world is crying out for sustainable clean energy sources.^1–3^ Hydrogen, which can generate electricity *via* fuel cells with nearly no emission of pollutants, is regarded as one of the most promising substitutes for fossil fuels.^4–6^ In order to store and transfer hydrogen conveniently, efficiently and safely, hydrogen storage materials with a moderate operating temperature, low cost, good dynamics and high hydrogen storage density are urgently required.^7^ Among numerous materials, magnesium hydride (MgH_2_) with high mass hydrogen storage capacity (7.76 wt%), good reversibility, low cost and other outstanding performances (LIB anode) has attracted intense attention worldwide.^8–11^ Nevertheless, two challenges (stable thermodynamics and poor kinetics) still need to be conquered before the large scale application of MgH_2_.^12–14^ In the past few decades, extensive research has been conducted to overcome these challenges through diverse methods, such as catalyst doping,^15–20^ alloying^21–24^ and nanotechnology.^25–27^

According to previous reports, transition metals (TMs) and their compounds showed a quite effective influence on improving the hydrogen storage properties of MgH_2_.^28–31^ For example, Liu *et al.*^32^ synthesized a Co@CNT nanocatalyst by carbonizing zeolitic imidazolate framework-67 and doped it into MgH_2_. The experimental results showed that the onset temperature of MgH_2_ decreased to 267.8 °C with the addition of Co@CNTs and the dehydrogenation capacity of MgH_2_–Co@CNTs could reach 6.89 wt% at 300 °C within 15 min. For absorption, the MgH_2_–Co@CNTs could absorb 6.15 wt% H_2_ at 250 °C within 2 min. Cheng *et al.*^33^ found that Pd_30_Ni_70_/CMK-3 could significantly improve the de/re-hydrogenation performance of MgH_2_ at low temperature. About 6 wt% hydrogen could be released below 290 °C and 4 wt% hydrogen could be absorbed at 70 °C under a hydrogen pressure of 3 MPa within 18 000 s. Besides pure metal, metal oxides, which can be easily synthesized, are preferred by scientists to improve the hydrogen storage performance of MgH_2_.^34–38^ Chen *et al.*^39^ found that a MgH_2_–Co/TiO_2_ composite started to desorb hydrogen at about 190 °C with a low apparent activation energy of 77 kJ mol^−1^. In addition, the dehydrogenated sample could absorb about 4.24 and 1.0 wt% hydrogen within 10 min at 100 and 50 °C, respectively. Bhatnagar *et al.*^40^ demonstrated that MgH_2_ catalyzed by Fe_3_O_4_@GS offers improved hydrogen storage behaviour. The MgH_2_–Fe_3_O_4_@GS had an onset desorption temperature of about 262 °C and the dehydrogenated sample could absorb 6.20 wt% hydrogen in 2.5 min under 15 atm H_2_ pressure at 290 °C. Mustafa *et al.*^41^ discovered that a MgH_2_-5 wt% CeO_2_ sample released about 3.6 wt% hydrogen in 30 min at 320 °C and the dehydrogenated sample could absorb approximately 3.95 wt% hydrogen within 5 min at 320 °C.

Apparently, both transition metals and their oxides can remarkably improve the hydrogen storage properties of MgH_2_. In our recent study,^42^ ZrMn_2_ was found to strikingly improve the hydrogen storage properties of MgH_2_; however, research on the catalytic effect of Mn based compounds on MgH_2_ has rarely been reported in the literature. In this work, Mn_3_O_4_ nanoparticles were successfully synthesized *via* a simple chemical method and adopted to enhance the comprehensive hydrogen storage properties of MgH_2_. To our knowledge, no studies have been conducted on doping Mn_3_O_4_ as a catalyst into MgH_2_. Further, the significantly improved hydrogenation and dehydrogenation performance of MgH_2_ catalyzed by Mn_3_O_4_ was systematically studied and the catalytic mechanism was also explored and discussed in detail.

## 2. Experimental

### 2.1 Sample preparation

All the chemical reagents were of analytical grade. The nano-Mn_3_O_4_ powder was prepared by decomposition of Mn(Ac)_2_·4H_2_O in diethylene glycol (DEG). Firstly, 2.45 g Mn(Ac)_2_·4H_2_O was dissolved into 100 ml DEG at a temperature of 80 °C. After this, the solution was heated to 160 °C in an oil bath pot and then kept for 8 h. After cooling to room temperature, the suspension was centrifuged and washed with deionized water and ethanol to remove the residual organic solvent. Finally, Mn_3_O_4_ nanoparticles (nano-Mn_3_O_4_) can be obtained after vacuum-drying at 70 °C for 10 hours.

MgH_2_ was prepared in our laboratory. The magnesium (purity 99.99%) used was purchased from Sinopharm Chemical Reagent. Mg powder was first hydrogenated at 380 °C and under a hydrogen pressure of 65 bar for 2 h. Then the sample was ball-milled at 450 rpm for 5 h and MgH_2_ was synthesized by repeating the above hydrogenation treatment. The nano-Mn_3_O_4_ powder and MgH_2_ with mass ratios of 5 : 95, 10 : 90 and 15 : 85 were mixed by ball milling. Ball milling of the above samples was performed on a QM-3SP4 planetary ball mill (Nanjing) at 400 rpm for 2 h under 1 bar of Ar (the ball to material ratio is 40 : 1). To avoid contamination and oxidation, all samples were handled and transferred in an Ar-filled glove box (Mikrouna) where the water/oxygen concentration was less than 0.1 ppm.

### 2.2 Sample characterization

The phase composition was analyzed by X-ray diffraction (XRD), which was carried out on an X'Pert Pro X-ray diffractometer (PANalytical, the Netherlands) with Cu K alpha radiation at 40 kV and 40 mA. A special container was used to prevent air and water contamination when samples were transferred and scanned. The morphology of samples was studied using scanning electron microscopy (SEM, Hitachi SU-70) and transmission electron microscopy (TEM, Tecnai G2 F20 STWIN) with energy dispersive spectroscopy (EDS). The hydrogen absorption and desorption results were obtained using Sieverts-type apparatus. Approximately 75 mg sample was heated to 430 °C at a heating rate of 2 °C min^−1^ in a stainless steel container during testing of the non-isothermal hydrogen desorption properties. Isothermal measurements were performed by quickly heating the sample to the target temperature and then keeping the temperature constant throughout the whole test. In addition, the isothermal absorption performance was measured at various temperatures under a hydrogen pressure of 30 bar while the isothermal desorption performance was tested at different temperatures under a hydrogen pressure below 0.01 bar.

### 2.3 Theoretical methods

All calculations were carried out within the DMol^3^ code.^43,44^ The generalized gradient approximation with the Perdew–Burke–Ernzerhof (PBE) functional was used to describe the exchange and correlation effects.^45^ The DFT semi-core pseudopod (DSPP) core treatment method was used for relativistic effects and replaces the core electrons with an effective potential and introduces a degree of relativistic correction into the core.^46^ The double numerical atomic orbital augmented by a polarization function (DNP) was chosen as the basis set.^47^ To achieve the calculation convergence, a smearing of 0.005 Ha (1 Ha = 27.21 eV) to the orbital occupation was applied. In the geometry structural optimization, the convergence tolerances of energy, maximum force and displacement were 1.0 × 10^−5^ Ha, 0.002 Ha Å^−1^ and 0.005 Å, respectively.

## 3. Results and discussion

### 3.1 Characterization of the prepared Mn_3_O_4_ nanoparticles

**Fig. 1:**
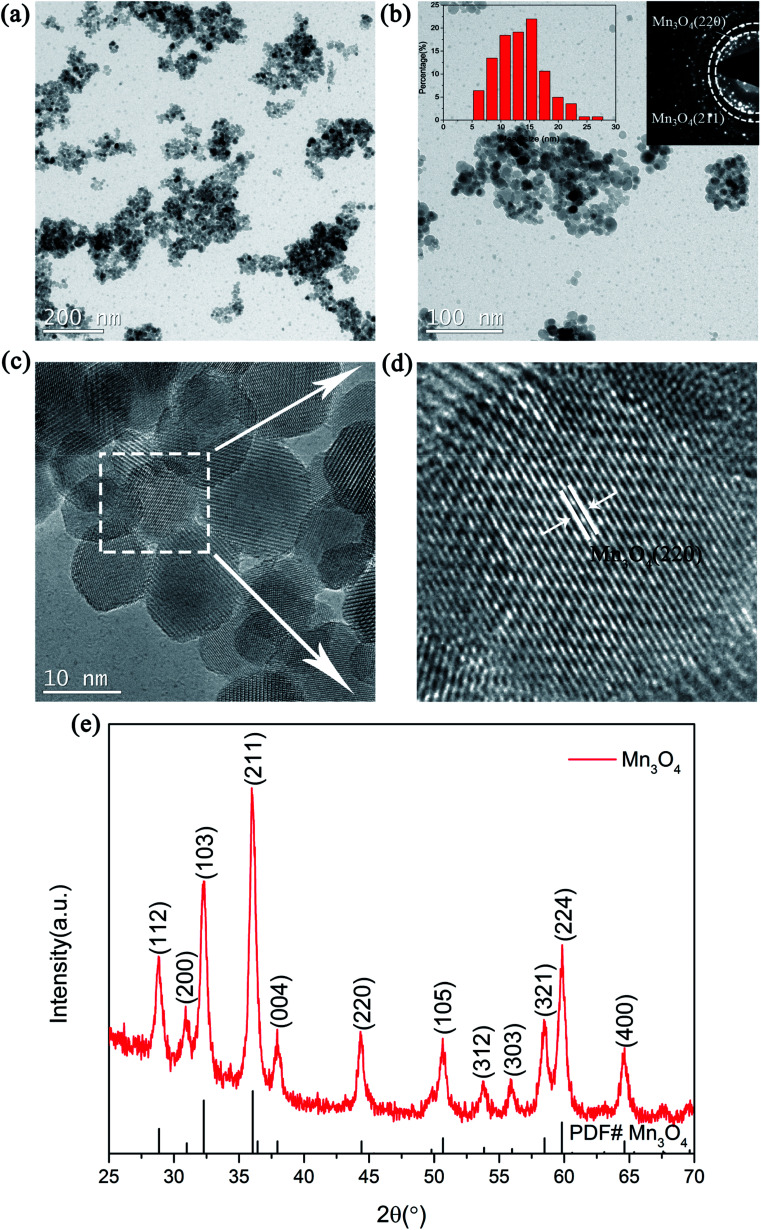
TEM images (a and c), TEM image with SAED patterns and particle size distribution curve (b), magnified TEM image (d) and XRD pattern (e) of the as-prepared Mn_3_O_4_ nanoparticles.

The structure and morphologies of the prepared Mn_3_O_4_ nanoparticles were analysed *via* XRD and TEM measurements, as shown in Fig. 1. Fig. 1a exhibits the TEM image of the Mn_3_O_4_ sample prepared by the simple chemical method. It can be clearly seen from the picture that the particle size of the as-prepared Mn_3_O_4_ ranged from 5 nm to 20 nm. Besides, the corresponding SAED image in Fig. 1b reveals that the diffraction rings correspond to the (211)/(220) of Mn_3_O_4_. In addition, it can be clearly seen from the particle size distribution curve that the size of prepared Mn_3_O_4_ was mainly concentrated in the range of 7 to 15 nm. The magnified TEM image of the marked part in Fig. 1c is shown in Fig. 1d, where the lattice planes corresponding to the (220) plane of Mn_3_O_4_ can be observed. The XRD examination result shown in Fig. 1e also indicates that the diffraction peak of Mn_3_O_4_ (PDF# 24-0734) can be clearly identified. Based on the TEM and XRD results, Mn_3_O_4_ nanoparticles were successfully synthesized and an outstanding catalytic effect on improving the hydrogen storage properties of MgH_2_ was expected.

### 3.2 Catalytic effect of the Mn_3_O_4_ nanoparticles on the hydrogen storage properties of MgH_2_

**Fig. 2:**
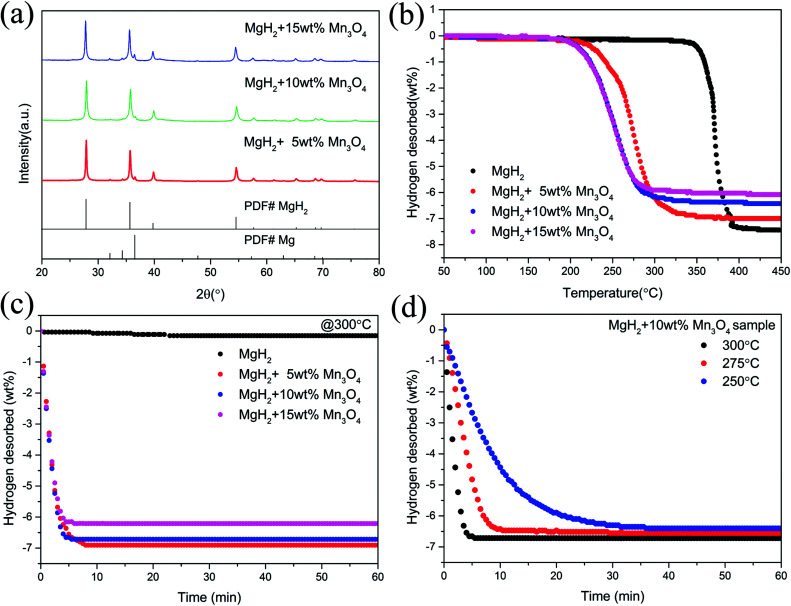
XRD patterns (a), volumetric release curves (b), and isothermal dehydrogenation curves (c and d) of MgH_2_, MgH_2_ + 5 wt% Mn_3_O_4_, MgH_2_ + 10 wt% Mn_3_O_4_, and MgH_2_ + 15 wt% Mn_3_O_4_ samples.

To examine the catalytic effect of Mn_3_O_4_ on the hydrogen desorption properties of MgH_2_, various amounts of Mn_3_O_4_ (5, 10 and 15 wt%) were doped into the MgH_2_ powders by ball-milling under a 1 bar Ar atmosphere for 2 h. The composites composed of MgH_2_ and *x* wt% Mn_3_O_4_ nanoparticles (*x* = 5, 10 and 15) were denoted as MgH_2_ + 5 wt% Mn_3_O_4_, MgH_2_ + 10 wt% Mn_3_O_4_ and MgH_2_ + 15 wt% Mn_3_O_4_, respectively. All of these milled composites were collected for structural characterization and property tests. Fig. 2a exhibits the XRD patterns of the Mn_3_O_4_-doped composites. Clearly, the MgH_2_ phase still dominated the XRD profiles and no obvious reacted phases occurred after ball milling. In addition, quite weak peaks of Mg can be seen in this pattern, which could be attributed to the unreacted Mg during synthesis.^15^ TPD (Temperature Programmed Desorption) analyses of different amounts of Mn_3_O_4_ nanoparticle doped MgH_2_ samples were also performed. Fig. 2b depicts the TPD curves of the above three composites and undoped MgH_2_ sample, revealing a single step of hydrogen release. It can be observed that the volumetric release curves of MgH_2_ + Mn_3_O_4_ composites shifted towards lower temperatures with the increasing added amount of Mn_3_O_4_. The as-synthesized MgH_2_ began to desorb hydrogen at 340 °C and released about 7.4 wt% hydrogen. With the addition of Mn_3_O_4_ nanoparticles, the initial desorption temperatures of the MgH_2_ + 5 wt% Mn_3_O_4_, MgH_2_ + 10 wt% Mn_3_O_4_ and MgH_2_ + 15 wt% Mn_3_O_4_ composites decreased to 230 °C, 200 °C and 200 °C, respectively. To further explore the hydrogen storage properties of the modified MgH_2_ systems, isothermal desorption measurements were carried out at 300 °C, as shown in Fig. 2c. The results show that the doping of Mn_3_O_4_ could significantly improve the hydrogen desorption kinetics of MgH_2_. The MgH_2_ + 5 wt% Mn_3_O_4_, MgH_2_ + 10 wt% Mn_3_O_4_ and MgH_2_ + 15 wt% Mn_3_O_4_ composites could desorb 6.9 wt%, 6.7 wt% and 6.2 wt% hydrogen in 8 min, while the pure MgH_2_ released only 0.038 wt% H_2_ in the same duration. From a comprehensive perspective of the dehydrogenation temperature and capacity, the MgH_2_ + 10 wt% Mn_3_O_4_ composite was chosen for further study. Fig. 2d illustrates the isothermal desorption profiles of the MgH_2_ + 10 wt% Mn_3_O_4_ composite at different temperatures (250, 275 and 300 °C). The composite could release 6.4 wt% hydrogen (nearly 94% of the theoretical hydrogen storage capacity) within 10 min at 275 °C and about 6.3 wt% hydrogen could be desorbed in 30 min even at 250 °C.

**Fig. 3:**
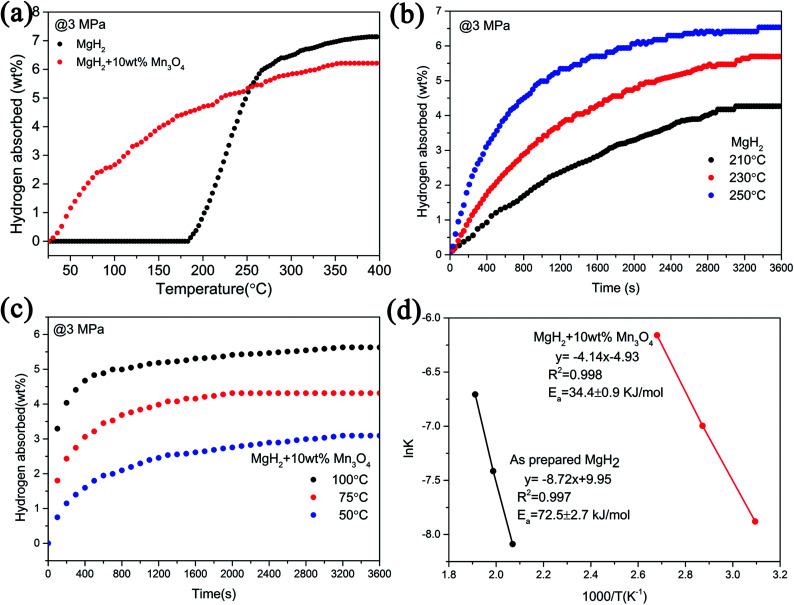
Non-isothermal hydrogenation curves (a), isothermal hydrogenation curves (b and c) and the corresponding Arrhenius plots (d) of MgH_2_ with and without 10 wt% Mn_3_O_4_.

Apart from the significantly improved desorption performance, we also focused on the absorption behaviour of the MgH_2_ + 10 wt% Mn_3_O_4_ composite. The isothermal and non-isothermal hydrogen absorption curves are shown in Fig. 3. Fig. 3a depicts the non-isothermal hydrogenation curves of the prepared MgH_2_ and MgH_2_ + 10 wt% Mn_3_O_4_ samples. The dehydrogenated MgH_2_ + 10 wt% Mn_3_O_4_ sample started absorbing hydrogen from room temperature, and about 5.4 wt% hydrogen could be taken up before 250 °C. However, the dehydrogenated MgH_2_ dilatorily absorbed hydrogen from 183 °C, which was approximately 160 °C higher than that for the dehydrogenated MgH_2_ + 10 wt% Mn_3_O_4_. Further isothermal absorption measurements of the as-prepared MgH_2_ and the MgH_2_ + 10 wt% Mn_3_O_4_ composite were conducted and the results are presented in Fig. 3b and c. At 50 °C, the dehydrogenated MgH_2_ + 10 wt% Mn_3_O_4_ sample exhibited a hydrogen absorption capacity of 2.5 wt% within 20 min. When heated to 75 °C, the hydrogen uptake of the Mn_3_O_4_ containing sample amounted to 4.1 wt% in 20 min. Within an identical time period, the hydrogen absorption capacity was increased to 5.1 wt% when the temperature was increased to 100 °C. Obviously, the MgH_2_ + 10 wt% Mn_3_O_4_ sample showed faster hydrogen absorption kinetics than pure MgH_2_ (Fig. 3b). Besides, the *E*_a_ values of the hydrogen absorption reaction were calculated to further explore the improved hydrogenation kinetics. Fig. 3d reveals the isothermal hydrogenation data of MgH_2_ and the MgH_2_ + 10 wt% Mn_3_O_4_ composite simulated using the Johnson–Mehl–Avrami–Kolmogorov (JMAK) equation,^48,49^ which can be written as:

1 ln[−ln(1 − *α*)] = *n* ln *k* + *n* ln *t*

where *α* is the fraction of Mg transformed into MgH_2_ at a particular time, *k* is an effective kinetic parameter, and *n* is the Avrami exponent. The values of *n* and *n* ln *k* obtained by fitting the JMAK plots are shown in Fig. S1.† The *E*_a_ values for the hydrogenation reactions were calculated according to the Arrhenius equation:^50^

2 *k* = *A* exp(−*E*_a_/*RT*)

According to the plots in Fig. 3d, the calculated *E*_a_ value of the absorption process for the dehydrogenated MgH_2_ + 10 wt% Mn_3_O_4_ was 34.4 ± 0.9 kJ mol^−1^, which was much lower than that of the dehydrogenated MgH_2_ (72.5 ± 2.7 kJ mol^−1^) and other MgH_2_-based systems published recently.^15,16^ The *E*_a_ for hydrogenation was distinctly decreased by 52.6%, indicating that the energy barrier for the absorption of hydrogen is remarkably reduced due to the addition of Mn_3_O_4_, which is reasonably responsible for the enhanced hydrogenation kinetics of the MgH_2_ + 10 wt% Mn_3_O_4_ composite.

**Fig. 4:**
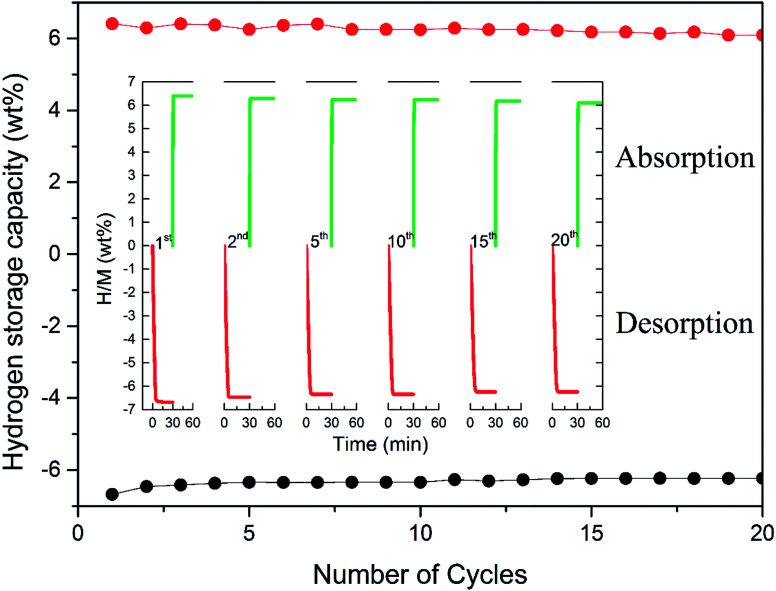
Non-isothermal dehydrogenation/hydrogenation curves and hydrogen storage capacity of the MgH_2_ + 10 wt% Mn_3_O_4_ composite.

To achieve the practical application of hydrogen storage materials, preserving long-term kinetics is considered one of the key technology indicators. Although favourable hydrogen absorption and desorption properties of Mn_3_O_4_ doped MgH_2_ were evidenced, the cycling performance of the MgH_2_–Mn_3_O_4_ composite still needs to be explored. The cycle behaviour of the MgH_2_ + 10 wt% Mn_3_O_4_ composite was tested at 300 °C for 20 cycles. As revealed in Fig. 4, the hydrogen storage capacity of the MgH_2_ + 10 wt% Mn_3_O_4_ composite reached 6.6 wt% in the first dehydrogenation process. When exposed to a 30 bar hydrogen atmosphere, the dehydrogenated sample could quickly absorb 6.4 wt% H_2_ at 300 °C. After 20 cycles, a high reversible capacity of 6.1 wt% was still maintained, which corresponds to 95.3% of the original capacity. Generally, MgH_2_ particles tend to grow and aggregate during pyrolysis, leading to the degradation of cycling performance.^51,52^ In one of our previous studies,^15^ the cycling results showed that the capacity of Fe doped MgH_2_ was obviously decreased after 20 cycles. It is evident that the hydrogen storage capacity of the MgH_2_ + 10 wt% Mn_3_O_4_ composite remained stable without significant decline after 20 cycles, revealing remarkable enhancement due to the catalytic activity of Mn_3_O_4_ nanoparticles.

### 3.3 De/hydrogenation mechanism

**Fig. 5:**
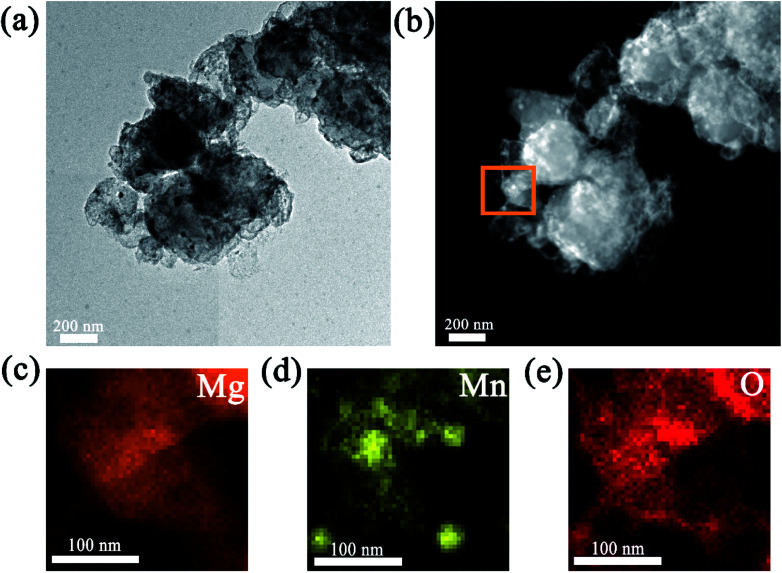
TEM image (a); STEM-HAADF image (b); corresponding EDS maps (c, d and e) of the MgH_2_ + 10 wt% Mn_3_O_4_ composite after 20 cycles.

As mentioned above, Mn_3_O_4_ showed a superior catalytic effect on improving the hydrogen absorption and desorption properties of MgH_2_. For a better understanding of the catalytic mechanism of the Mn_3_O_4_ modified MgH_2_ system, a deeper investigation was conducted. It can be seen in the TEM image (Fig. 5a) that the average size of the MgH_2_ + 10 wt% Mn_3_O_4_ composite after 20 cycles approached 300 nm, which is much smaller than that of ball-milled MgH_2_ (over 800 nm) as shown in Fig. S2.† Moreover, the HAADF-STEM image in Fig. 5b shows that numerous bright nanoparticles were homogeneously distributed on the surface of MgH_2_. Corresponding EDS mapping demonstrated that Mn and O covered almost the whole surface of the MgH_2_ + 10 wt% Mn_3_O_4_ composite after 20 cycles.^53^

**Fig. 6:**
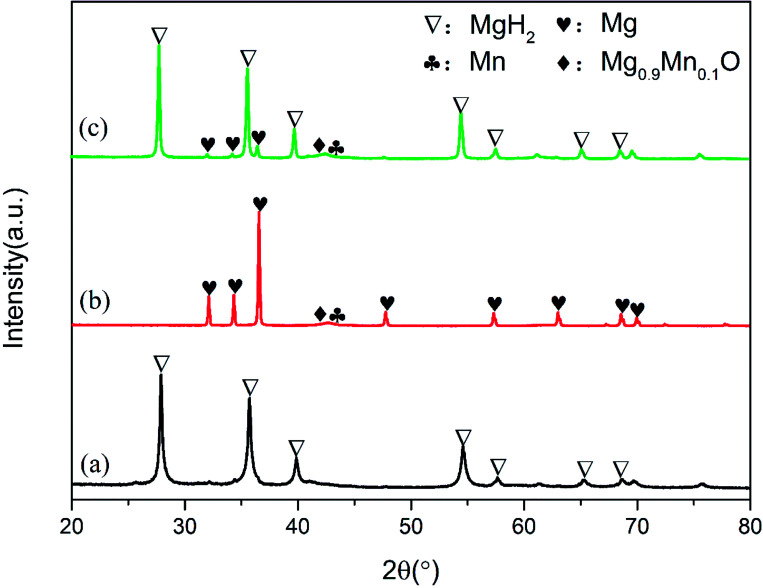
XRD patterns of ball-milled (a), dehydrogenated (b) and hydrogenated (c) MgH_2_ + 10 wt% Mn_3_O_4_ samples.

To elucidate the evolution of Mn_3_O_4_ nanoparticles in the de/hydrogenation process, the MgH_2_ + 10 wt% Mn_3_O_4_ sample in the ball-milled, dehydrogenated and re-hydrogenated states was collected and examined by XRD measurements (Fig. 6). Although the diffraction peaks of Mn_3_O_4_ were unclear in the XRD patterns, TEM measurements (Fig. S3†) demonstrated that small particles of Mn_3_O_4_ can be observed to be evenly distributed on the surface of MgH_2_. Compared with that of pure MgH_2_, the particle size of MgH_2_ + 10 wt% Mn_3_O_4_ in Fig. S3† was much smaller. Clearly, MgH_2_ or Mg still dominated the XRD patterns in Fig. 6 after the doping of Mn_3_O_4_. However, it is interesting that Mn and Mg_0.9_Mn_0.1_O phases occurred in both the dehydrogenated and rehydrogenated samples, as shown in Fig. 6b and c. In addition, the TEM image (Fig. S4†) reveals that Mn particles were uniformly dispersed on the surface of MgH_2_. It should be pointed out that Mn_3_O_4_ in contact with Mg can readily form Mn and Mg_0.9_Mn_0.1_O under conditions of high temperature.^40^ The XRD and TEM results indicate that the added Mn_3_O_4_ nanoparticles were reduced to metallic Mn during the de/hydrogenation process. Therefore, we believe that metallic Mn is the key active catalytic species which enhance the hydrogen release/uptake for MgH_2_/Mg.

**Fig. 7:**
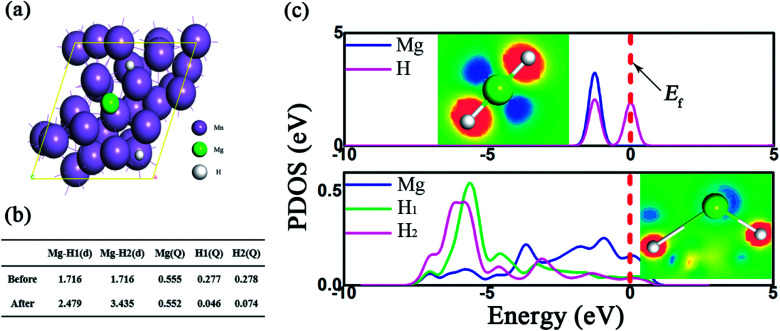
MgH_2_ absorption on the surface of Mn (330). The absorption configuration (a), the bond length, the Mulliken population of Mg–H before and after absorption on the Mn (330) surface (b) and the corresponding partial density of states (c). Inset: the deformation density of MgH_2_ where blue or orange denotes the charge depletion or accumulation.

The impact of Mn metal on the catalytic performance was further investigated by DFT calculations, where the Mn (330) surface was modeled, as shown in the TEM picture. The bond length and the Mulliken population (MP) are described in Fig. 7b. As presented, due to the presence of the Mn support, the Mg–H bonds were dramatically elongated and the average bond length is 2.48 Å, much longer than the pristine one (1.72 Å). Besides, the partial density of states (PDOS) in Fig. 7c shows that the ss-orbital overlap of the Mg–H bond was obviously weakened and the s electrons of Mg and H were mainly distributed at −5 ∼ 0 eV and −5 ∼ −6 eV, respectively. This reduced orbital interaction corresponds to the faint bond strength, which benefits the de/hydrogenation kinetics.^54^ Therefore, the presence of Mn plays an important role in improving the hydrogen storage properties of the MgH_2_ molecule.

**Fig. 8:**
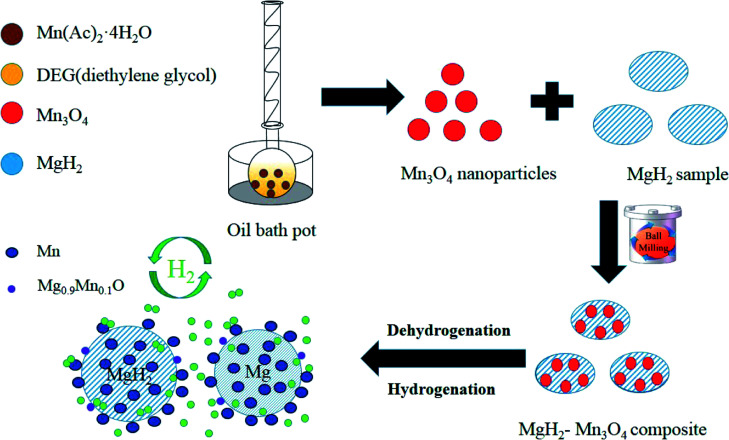
Schematic summary of the synthesis and de/hydrogenation process in MgH_2_–Mn_3_O_4_ composites.

Based on the XRD, TEM and calculation results, the whole synthesis and de/hydrogenation process is illustrated in Fig. 8. Mn_3_O_4_ nanoparticles prepared by a facile chemical method were evenly decorated on the surface of MgH_2_ during ball milling. In the de/hydrogenation process, Mn_3_O_4_ was reduced to metallic Mn. The Mn “coating”, on one hand, will promote the fracture of the Mg–H bond in MgH_2_ and reduce the de/hydrogenation temperature. On the other hand, it will help to separate the MgH_2_ particles and prevent them from growing and aggregating, thus preserving the stable cycling properties. As a result, the *E*_a_ value of the hydrogen absorption reaction was greatly decreased and the hydrogen storage properties of the Mn_3_O_4_-doped MgH_2_ were significantly enhanced.

## 4. Conclusion

In summary, Mn_3_O_4_ nanoparticles around 10 nm were successfully synthesized by a simple chemical method and a series of experiments proved that Mn_3_O_4_ can remarkably improve the hydrogen storage properties of MgH_2_. During the process of the non-isothermal mode, the MgH_2_ + 10 wt% Mn_3_O_4_ composite released approximately 6.4 wt% hydrogen from 200 °C to 300 °C. Moreover, the MgH_2_ + 10 wt% Mn_3_O_4_ composite desorbed 6.7 wt% hydrogen within 8 min under the isothermal conditions of 300 °C. The dehydrogenated MgH_2_ + 10 wt% Mn_3_O_4_ sample could absorb hydrogen under 3 MPa H_2_ pressure even at room temperature, and about 5.4 wt% hydrogen could be charged before 250 °C. The *E*_a_ of rehydrogenation of the MgH_2_-10 wt% Mn_3_O_4_ composite was calculated to be 34.4 ± 0.9 kJ mol^−1^. In addition, this sample exhibited favourable cycling stability with no significant fading over 20 cycles. Further XRD, TEM and theoretical calculations revealed that the *in situ* formed Mn weakened the Mg–H bond, and thus greatly promoted the de/hydrogenation reaction and preserved the cycling performance. This study combined experimental results with DFT calculations to investigate the catalytic effect of Mn_3_O_4_ on improving the hydrogen storage properties of MgH_2_, which will be a good reference for developing a new composite hydrogen storage system.

## Conflicts of interest

There are no conflicts to declare.

## Supplementary Material

NA-002-D0NA00137F-s001
