# Supplementary material for: Excellent catalysis of Mn3O4 nanoparticles on the hydrogen storage properties of MgH2: an experimental and theoretical study
Source: Nanoscale Adv. 2020 Mar 9;2(4):1666–75. doi: 10.1039/d0na00137f (PMC9418404; doi:10.1039/d0na00137f)
Supplement: NA-002-D0NA00137F-s001 [file NA-002-D0NA00137F-s001.pdf]

Electronic Supplementary Material (ESI) for Nanoscale Advances.
This journal is © The Royal Society of Chemistry 2020

Electronic Supplementary Material

Excellent catalysis of Mn_3O_4 nanoparticles on the hydrogen storage properties of MgH_2: An experimental and theoretical study

Liuting Zhang[a, §], Ze Sun[a, §], Zhendong Yao[a, §], Lei Yang[a], Nianhua Yan[a], Xiong Lu[a], Beibei Xiao[a], Xinqiao Zhu[b,*] and Lixin Chen[c,*]

[a] *College of Energy and Power, Jiangsu University of Science and Technology, Zhenjiang 212003, PR. China*

[b] *Institute of Nuclear Physics and Chemistry, China Academy of Engineering Physics, Mianyang 621999, PR. China*

[c] *State Key Laboratory of Silicon Materials, Department of Materials Science and Engineering, Zhejiang University, Hangzhou 310027, PR. China*

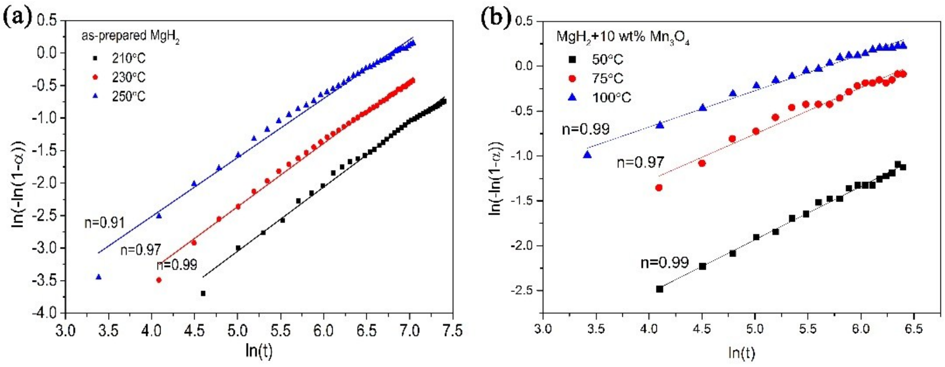

Fig.S1 JMAK plots of MgH_2 and MgH_2+10 wt% Mn_3O_4 composite.

* To whom correspondence should be addressed. Tel: +86 17738406685; E-mail address: zhuxinqiao@zju.edu.cn, lxchen@zju.edu.cn

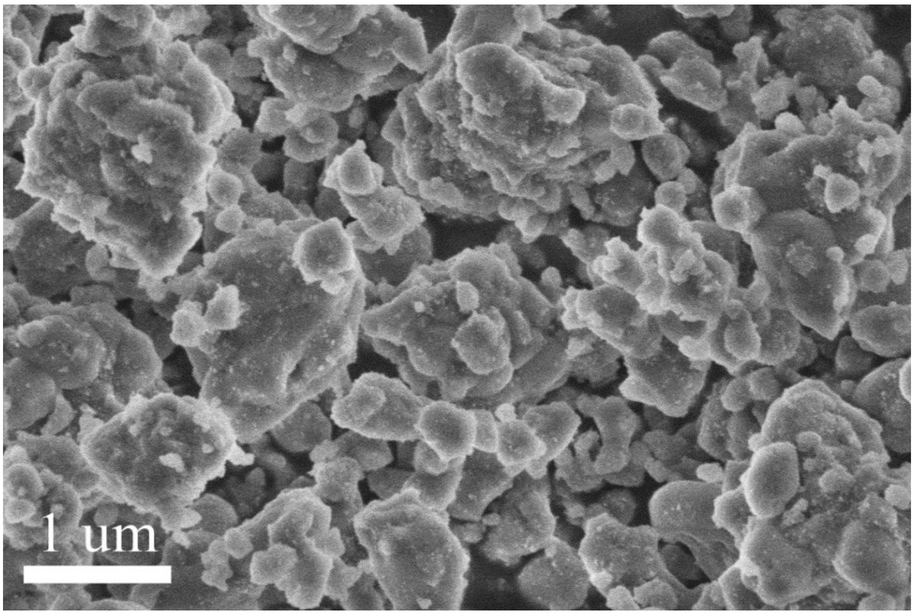

Fig.S2 SEM image of as-prepared MgH_2.

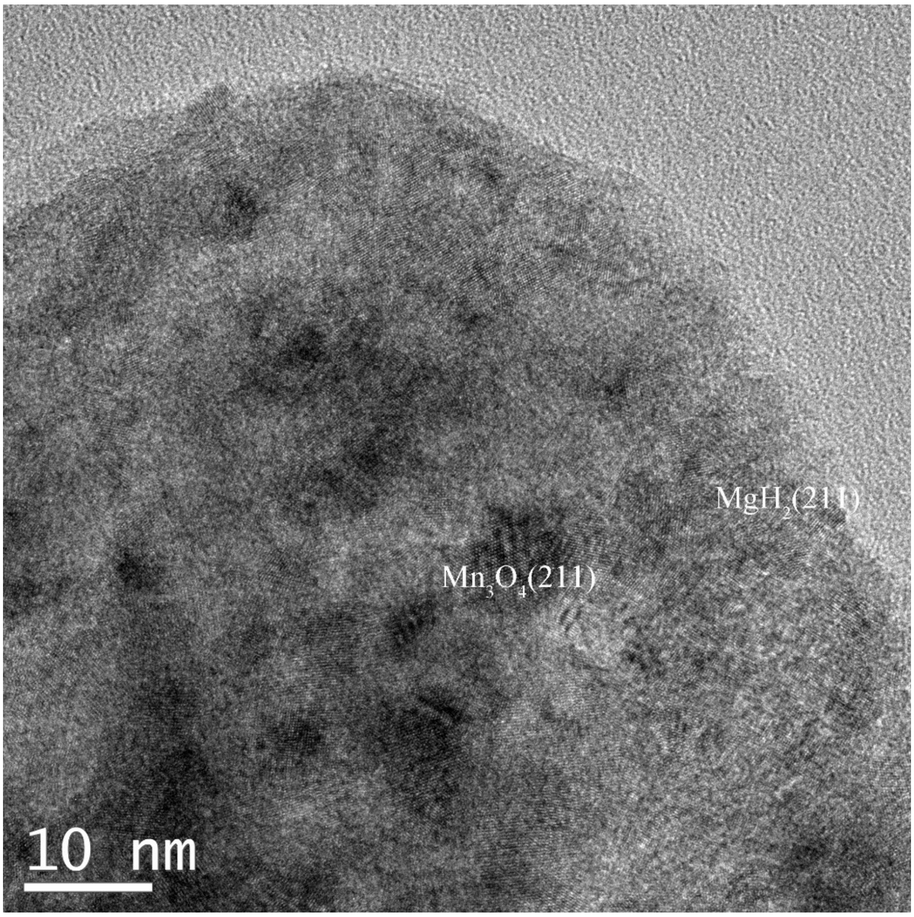

Fig.S3 TEM image of MgH_2+10 wt% Mn_3O_4 composite in ball-milling state.

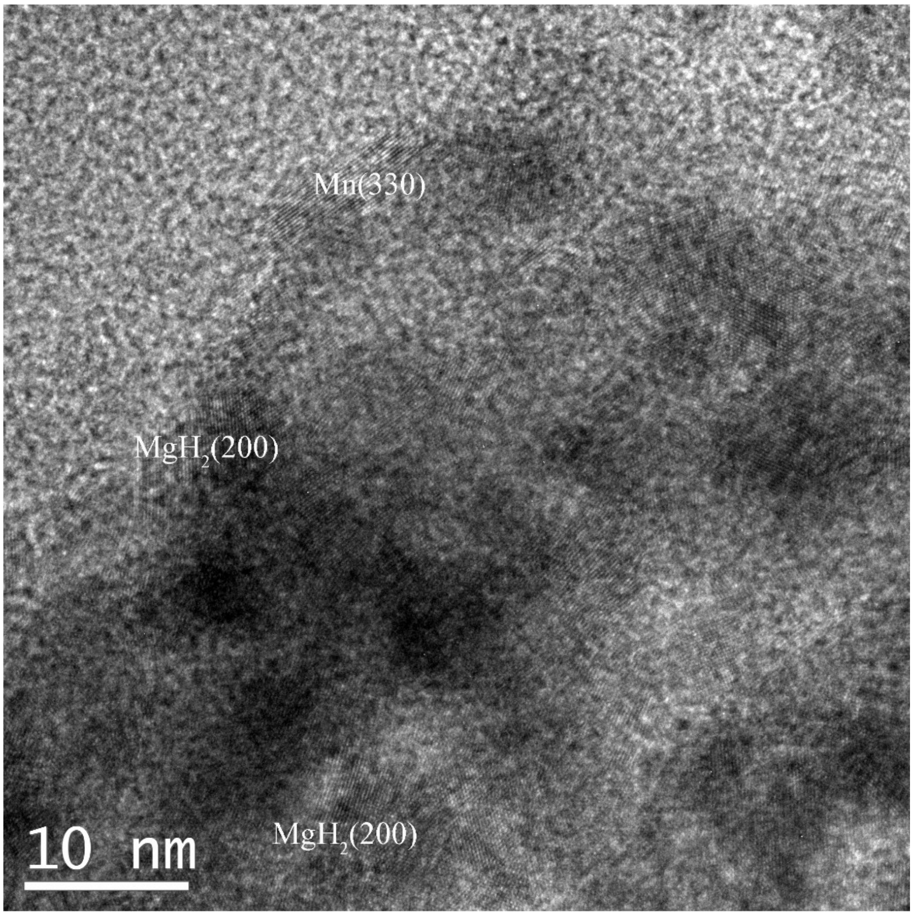

Fig.S4 TEM image of MgH_2+10 wt% Mn_3O_4 composite after 20 cycles.
